# Supplementary material for: Oxidative Stress and Chemoradiation-Induced Oral Mucositis: A Scoping Review of In Vitro, In Vivo and Clinical Studies
Source: Int J Mol Sci. 2022 Apr 27;23(9):4863. doi: 10.3390/ijms23094863 (PMC9101413; doi:10.3390/ijms23094863)
Supplement: Supplementary file 1 [file ijms-23-04863-s001.zip › ijms-1671515-supplementary.pdf]

Supplementary Materials

In Vitro Studies

Supplementary Table S1. Summary of in vitro study design and results.

AUTHOR, YEAR	POPULATION	INTERVENTION	COMPARATOR	OUTCOME	EFFECT OBSERVED	MECHANISM OF ACTION
Osaki et al., 1994 [6]	Sheep red blood cells (SRBC; 5*10^6/ml)	Incubated with various concentration of Azelastine (3,30,300ng/ml, 3, 30ug/ml)	No Azelastine	Cell viability	Azelastine suppressed neutrophil respiratory burst in vitro $p < 0.05$.	
Greenberger et al., 2014 [7]	Bone marrow cultures were established from Fancd2−/−, Fancd2+/−, and Fancd2+/+ mice (C57BL/6J mouse strain)	GS-nitroxide, JP4-039 added 1 hr biradiated to doses between 0 and 800 cGy before or immediately after irradiation, maintained in culture plate for 7 days.	No radiation	DNA double stranded breaks, antioxidant	Decreased production of hematopoietic cells by cultures over 22 weeks.	
Yamaguchi et al., 2019 [8]	Human dermal fibroblast cell line	Astaxanthin (5µM) treatment prior to cisplatin exposure	Cisplatin with 0.05% dimethyl sulfoxide	ROS production reduced, seen by flow cytometry	AST reduces damage from cisplatin, ROS generation. $p < 0.05$.	AST is a strong ROS scavenger, inhibits UV-A effect on ROS directed signalling cascade.
Vaillancourt et al., 2021 [9]	Oral keratinocyte cell line insulted by irinotecan	Green tea extract (polyphenol content ≥ 98 %, including 45 % EGCG) EGCG	No intervention	ROS production by fluorometric assay	Treatment led to reduced ROS production compared to untreated cells ($p <$ 0.001).	Polyphenols works as effective ROS scavenging agents.
Iglesias-Bartolome et al., 2012 [10]	Irradiated normal oral keratinocyte cell culture	Rapamycin	HN12 and Cal27 (+); untreated or vehicle (-)	Levels of ROS after radiation in NOK and HN12 cells with rapamycin treatment	Rapamycin increased the protein levels of MnSOD in NOKs.	Rapamycin increases MnSOD expression resulting protection from oxidative stress.

Baek et al., 2014 [11]	Human keratinocyte (HaCaT) cells	3-amino-3-(4-fluoro-phenyl)-1H-quinoline-2,4-dione (KR22332)	HaCaT cells with radiation therapy only without KR22332	Intracellular ROS generated by DCFDA	KR22332 inhibits radiation-induced intracellular ROS generation $p < 0.05$.	KR22332 inhibits radiation-induced intracellular ROS generation.
Chang et al., 2014 [12]	Human keratinocyte (HaCaT) cells	Various concentration of Korean red ginseng (KRG)	No KRG	Intracellular ROS generation detected using flow cytometry with, DCFDA	KRG significantly inhibited the intracellular ROS generation induced by irradiation $p < 0.01$.	MMP and KRG decreased the level of ROS leading to prevention of radiation-induced apoptosis of HaCaT
Lambros et al., 2014 [13]	Human buccal keratinocytes	1 mM NAC, 5 mg/mL QYD, or an NAC-QYD mixture (1 mM NAC + 4.5 mg/mL QYD)	Untreated 3D tissues	Cell viability	NAC-QYD treatment in tissues led to preservation of their integrity and prevention of apoptosis. $p < 0.05$.	Significant increase of metallothioneins, HMOX1, and other genes of the Nrf2 pathway, which protect cells against oxidative stress were seen in QYD-treated tissues.
Maiguma et al., 2009 [14]	Normal human epidermal keratinocytes and normal human periodontal ligament fibroblasts	Amifostine and Cyclooxygenase-1 Inhibitor	Control without chemical	Cell viability and superoxide radical activity	Allopurinol was a superoxide radical scavenger, while amifostine was hydroxyl radical scavenger $p < 0.05$.	In concentration-dependent manner, amifostine have hydroxyl radical scavenging activity and weak superoxide radical scavenging activity.
Shin et al., 2013 [15]	Human keratinocyte line HaCaT	Pre-treated with various concentrations of Epicatechin (0–200 μM) for 1 hr	No EC	ROS production - flow cytometry	EC significantly reduced radiation-induced the generation of intracellular ROS generation $p < 0.01$.	
Tsubaki et al., 2018 [16]	Primary normal human oral keratinocytes (NHOK cells)	Treated with rebamipide (0.01–0.5 mM) in combination with 5-FU (1–50 μM) or cisplatin (0.5–5 μM)	Rebamipide	Effects of SB203580 and U0126 on rebamipide-induced cytoprotection in NHOK cells, number of dead cells was counted	LY294002 or rapamycin prevented rebamipide-induced Bcl-2 and Bcl-xL expressions, and decreased Bax and Bim expression $p < 0.01$.	Rebamipide activates Akt and mTOR. By suppressing growth arrest DNA damage-induced 45α expression, ROS-mediated mitochondrial damage, and caspase activation in RGM1

						cells, rebamipide prevents indomethacin-induced apoptosis
Kim et al., 2020 [17]	Human immortalized keratinocytes, HaCaT cells	Pre-treatment with NAC (10 mM) for a 1 hour before 10 minutes of radiation with 6 MV LINAC		ROS production	Radiotherapy-induced ROS generation was significantly reduced by NAC $p < 0.05$.	NAC treatment prevents radiation-induced mucositis by decreasing NRF2-dependent ROS synthesis.
Takano et. al, 2014 [18]	RT7, an immortalized human oral keratinocyte cell line	γ-tocotrienol 10nM	No γ-tocotrienol	Measurement of ROS, Expression of HO-1 (heme oxygenase)	γ-tocotrienol could prevent 5-FU-induced ROS generation $p < 0.01$.	γ-tocotrienol led to stabilization of Nrf2 activation, which led to ROS detoxification and high cell survival rate.
Cirillo et al., 2015 [19]	Normal human oral fibroblasts (NHOF), Keratinocytes	Keratinocytes treated with H_2O_2 or TGF-β, with or without Mucosamin (hyaluronic acid-based compound)	No Mucosamin, no H_2O_2	Cell viability	Mucosamin1showed a protective effect oxidative stress-induced senescence.	Binding of high molecular mass HA to free radicals catalyses the breakdown of HA into smaller fragments and thus inactivates ROS.
Rupel et al., 2018 [20]	Human keratinocytes (HaCaT)	Exposed 100 ng/mL LPS for 15 minutes or not and then exposed to photomodulation at either 970 nm 800nm, 660 nm	No LPS, No PBM	Intracellular ROS Production and Kinetics in PMN	Both 970 nm protocol and the 800 nm protocol led to significant ROS reduction.	
Chung et al., 2009 [21]	IMR90 cells, derived from normal human embryonic fibroblasts	Treatment with 1 mM phenylbutyrate for 24 h	No phenylbutyrate	DNA damage repair and cell survival	Phenylbutyrate induced histone H3 hyperacetylation in the normal human fibroblasts $p < 0.01$.	HDAC inhibitor phenylbutyrate remodels chromatin structure leading to double-strand damage repair.
Huth et al., 2020 [22]	Normal human dermal fibroblasts (NHDF) and normal human epidermal keratinocytes (NHEK) as well as normal	Treated with expanthenol-containing ointment (DCO)	Placebo-treated and untreated controls	ROS production	Addition of calcium pantothenate led to significant reduction in ROS production.	

	human oral mucosa keratinocytes (NHOK) and normal human oral mucosa fibroblasts (NHOF)					
Yoshida et al., 2014 [23]	Sa3 cells (gingival cells)	Incubated at different concentration of 5-FU and TJ84 (Daiokanzoto) 24 h	No TJ84	Mitochondria-specific ROS	TJ-84 reduced the chemotherapy-induced mitochondria-derived-O_2, compared to the control cells $p < 0.01$.	
Shinde et al., 2016 [24]	TC-1 epithelial cell from Fancd2+/+, Fancd2+/− and Fancd2−/− mice	1 hour treatment of 10 *µM* JP4-039 before irradiation, or immediate addition of JP4-039 was after irradiation	Without JP4-039		Mitochondrial targeting makes F15/JP4-039 an effective protector from radiation. F15/JP4-039 can reduce radiotherapy-induced OM.	
Kim et al., 2017 [25]	Human pharyngeal cell (Detroit 562, ATCC CCL-138)	Salvia miltiorrhiza Bunge (SM) on 5-fluorouracil (5-FU)-treated human pharyngeal cells.	No SM	ROS production (ROS assay)	Intracellular ROS production was decreased with SM treatment and DPPH-scavenging activity was increased. $p < 0.001$.	SM treatment led to proliferation and protection from 5-FU-induced cytotoxicity.
Park et al., 2018 [26]	Human pharyngeal cell line Detroit 562 (ATCC CCL-138)	OCE treatment	Treated with each concentration of OCE (1, 5, 10, 50, or 100 µg/mL) or with each concentration of OCE plus 10 µM 5-FU	Intracellular ROS production	OCE decreases intracellular ROS production in human pharyngeal cell line $p < 0.01$.	Through MTT assay, DPPH radical scavenging activity, and ROS production, increased cell viability was observed due to high antioxidant activity of OCE.

In Vivo Studies

Supplementary Table S2. Summary of *in vivo* study design and results of various chemical compounds as interventions.

AUTHOR, YEAR	POPULATION	INTERVENTION	COMPARATOR	OUTCOME	EFFECT OBSERVED	MECHANISM OF ACTION
Iglesias-Bartolome et al., 2012 [10]	Rat model of radiotherapy-induced OM	Rapamycin<br>*N* = Unspecified, at least 6	Untreated<br>*N* = Unspecified, at least 6	Relative ulceration evaluation<br><br>Histological analysis of tongue<br><br>γH2AX and mitochondrial SOD assay	Rapamycin treated rats were completely protected (no *p*-value provided).<br><br>Histology of rat tongue after rapamycin showed preservation of epithelial layer after radiation.<br><br>p53, γH2AX expression was reduced and mitochondrial SOD level increased in rapamycin treatment compared to untreated (no *p*-value provided).	Increased mitochondrial SOD expression suggestive of promoting antioxidant effect and protection from radiation-induced OM and ulcers.
Shin et al., 2013 [15]	Female Sprague-Dawley rats of six weeks old.	Administration of various concentrations of Epicatechin (up to 200 mM) after the radiation therapy.<br>*N* = 8 each	Control group did not receive any radiation or Epicatechin, and the radiation-only group received radiation therapy only.<br>*N* = 8 each	Intracellular ROS production and cell viability were measured to see the protective effects of Epicatechin on the radiation-induced oral mucositis.<br><br>Histological analysis	Epicatechin administration led to significant reduction in intracellular ROS generation ($p < 0.01$) and prevented cell apoptosis induced by radiation therapy ($p < 0.01$).<br><br>Epicatechin administration also saw reduction in oral mucosa ulceration and necrosis.	Reduced intracellular ROS associated with reduced cell apoptosis and OM severity following radiation therapy
Kim et al., 2020 [17]	Six-week-old female Sprague-Dawley rats	N-acetylcysteine (NAC) treatment (airflow of 10.01L/min for 5 minutes) after radiotherapy<br>*N* = 10	Saline treatment after radiotherapy<br>*N* = 10	ROS generation and protein-related DNA damage measured to evaluate protective effect of NAC against	NAC led to significant reduction in the ROS production and showed preventive effect against radiation-induced oral mucositis ($p < 0.001$).	Suggestive that NAC inhibits ROS production mechanism for OM prevention

				radiotherapy-induced oral mucositis.		
Ara et al., 2008 [27]	Five – six-week-old male golden Syrian hamsters	Intraperitoneal injection of velafermin (4mg/kg) diluted into vehicle (7% Aminosyn). $N = 10$	Administration of the vehicle only (7% Aminosyn). $N = 10$	Visual examination of oral mucositis<br><br>Inflammatory cytokines (IL-6), and NF-E2-related factor-2 were measured to see the protective effects of velafermin against radiotherapy-induced oral mucositis.	Administration of velafermin led to significant reduction of the degree of oral mucositis compared to the control group ($p < 0.001$).<br><br>Velafermin treatment led to significant reduction in IL-6 level and led to significant increase in the NF-E2-related factor-2 (increased protection from ROS) ($p < 0.001$).	IL-6 pro-inflammatory cytokine and NF-E2-related factor-2, involved in DNA repair, increased and was associated with improvement in OM condition. However, no oxidative stress mechanism directly evaluated
Clemenson et al., 2019 [28]	Female C57BL/6 mice.	Slow tail vein injection of CPh-1014 (free radical scavenger). $N = 6$	No injection of CPh-1014 (free radical scavenger). $N = 6$	DNA damage was evaluated, by assessment of γH2AX foci formation, to see the protected effect of CPh-1014 against radiation-induced oral mucositis.	CPh-1014 injection was able to reduce the radiation-induced DNA damage in oral mucosa and reduced the severity of oral mucositis ($p < 0.05$).	Reduced genetic damage but no reported antioxidant mechanism reported
Nakajima et al., 2015 [29]	Mouse model of radiotherapy-induced OM	30 or 300mg/kg edaravone $N = 20$ each	Control, intact $N = 20$ each	Survival rate<br><br>Mean and total OM score<br><br>Histopathological analysis<br><br>TUNEL assay<br><br>Lipid peroxidation by TBARS assay<br><br>MPO activity	Survival rate decreased after 12 days of initial treatment in both edaravone groups.<br><br>Edaravone groups had reduced mean OM score 10 and 12 days after initial treatment ($p < 0.05$).<br><br>Edaravone groups had lower total OM score compared to control ($p < 0.05$).	Reduction of lipid peroxidation and MPO associated with reduced OM severity

					Histopathologically, edaravone groups had limited epidermolysis and inflammatory cell infiltration.<br><br>TUNEL positive cells were lower in both edaravone groups ($p < 0.05$).<br><br>Both edaravone groups had reduced TBARS levels, but only 300mg/kg edaravone was statistically significant ($p < 0.05$).<br><br>MPO activity was reduced in both edaravone groups ($p < 0.05$).	
Mafra et al., 2019 [30]	Hamster model of chemotherapy-induced OM	1, 5 or 10mg/kg Gliclazide<br>$N = 5$ per group	Saline<br>$N = 5$	OM evaluation<br><br>Histopathological analysis and scoring<br><br>Oxidative stress by MDA and MPO assays<br><br>Inflammation by ELISA assay	Reduced OM score at 1, 5 and 10mg/kg gliclazide ($p < 0.05$; $p < 0.05$; $p < 0.01$, respectively).<br><br>10mg/kg gliclazide had a lower histopathological score compared to saline treated mice ($p < 0.01$).<br><br>10mg/kg gliclazide reduced MDA and MPO levels ($p < 0.05$); and TNF-α and IL-1β levels ($p < 0.05$).	MDA and MPO reduction suggestive of limiting OM severity via oxidative stress mechanism.
Yang et al., 2018 [31]	Mouse model of radiotherapy-induced OM	4 or 6g/kg DMSO by i.p<br>$N = 7$	Saline<br>$N = 7$<br><br>Untreated<br>N=7<br><br>rhKGF<br>$N = 7$	Histological evaluation<br><br>Thickness of tongue mucosa<br><br>P63+ cell density<br><br>Ki-67+ cell density	DMSO completely resolved inflammation induced by radiation<br><br>Tongue mucosa thickness increased compared to saline groups 7 ($p < 0.01$) and 9 ($p < 0.001$) days after irradiation. Mucosal thickness by DMSO was equivalent to amifostine and rhKGF	γ-H2AX has initial cytoprotective effect via DNA repair. However, oxidative stress mechanism not evaluated.

			Amifostine<br>$N = 7$	Immunofluorescence analysis	Ulcer size reduced when DMSO was injected 1 hour ($p < 0.001$), 3 hours ($p < 0.01$) and 6 hours ($p < 0.05$) prior to irradiation, when compared to saline. DMSO was more effective than amifostine or rhKGF in reducing ulcer size<br><br>DMSO treatment maintained proliferative potential of epithelial cells compared to vehicle controls, by p63+ and Ki-67+ quantification ($p < 0.05$)<br><br>After 6 hours of irradiation, DMSO reduced the proportion of cells positive for γ-H2AX.	
Ortiz et al., 2015 [32]	Male Wistar rats	Radiation therapy and application of gel with 3% melatonin or intraperitoneal injection of melatonin.<br>$N = 4\text{-}6$	Radiation therapy and administration of either gel with no melatonin or intraperitoneal injection of saline.<br>$N = 4\text{-}6$	LPO, GPx, GRd, and GSH/GSSG assayed by western blot<br><br>Histopathological analysis of oral tissue	GPx increased; GSSG/GSH, LPO and GRd decreased in melatonin treated groups ($p < 0.05$)<br><br>Melatonin administration reduced oral mucositis ulcer size ($p < 0.001$).	Reduced GSSG/GSH ratio, LPO and GRd associated with improved oral mucositis presentation
Shimamura et al., 2019 [33]	Mouse model of chemotherapy-induced OM	0.015 or 0.03μM GGsTop N=5 each	Saline<br>$N = 5$<br><br>Untreated<br>$N = 5$	Ulcer area<br><br>Treatment period<br><br>Histological analysis of oral mucosa	0.03μM GGsTop reduced ulcer area at day 5 and after ($p < 0.05$).<br><br>Mean treatment period for 0.03μM GGsTop, until resolution of OM, was significantly lower at 16 days compared to untreated group at 18 days ($p < 0.05$).	GGsTop selectively inhibits GGT to promote glutathione.

					Histologically, there was early oral mucosal healing in mice treated with 0.03µM GGsTop compared to untreated. Furthermore, collagen production was promoted in 0.03µM GGsTop group	
Im et al., 2019 [34]	Five – eight-week-old female C57BL/6 mice	Administration of various NecroX-7 dosage (0.3, 3 or 30 mg/kg) before 5-FU treatment. *N* = 8 each	Administration of saline solution before 5-FU treatment. *N* = 8	ROS and γH2AX quantified as main oxidative stress biomarkers,<br><br>Mitochondrial structure and HMGB1 translocation were examined	In the experimental groups, NecroX-7 administration led to reduced oxidative stress, dysfunction of mitochondria, and apoptosis in 5FU-induced oral mucositis. Levels of ROS and γH2AX have been reduced by NecroX-7 ($p < 0.001$).	Reduced ROS and γH2AX, implicated in chemotherapy-induced OM, and mitochondrial dysfunction
Ala et al., 2020 [35]	Thirty-three Wistar rats.	Daily dose administration of sumatriptan intraperitoneally for four consecutive days along with radiotherapy. *N* = 3-6 each	Daily saline administration intraperitoneally along with radiotherapy. *N* = 3-6 each	Malondialdehyde (MDA) and inflammatory cytokine (TNF-α) levels were measured to see the protective effect of sumatriptan against radiotherapy-induced oral mucositis.	Sumatriptan treated rats had well maintained oral epithelial thickness and reduced mucosal damage than untreated ($p < 0.05$).<br><br>There was a significant increase in the MDA level (marker of lipid peroxidation) and TNF-α in control group, compared to the healthy rats ($p < 0.0001$). There was a significant decrease in the MDA level and TNF-a in the experiment group with sumatriptan, compared to the control groups ($p < 0.01$).	Reduction in MDA was associated with the reduction in OM severity.
Yoshino et al., 2014 [36]	Hamster model of chemotherapy-induced OM	60mg/kg 5-FU + 10% acetic acid *N* = 8-11	Saline *N* = 8-11<br><br>10% acetic acid *N* = 8-11	Area of OM<br><br>MDA concentration	Mice treated with 5-FU alone was not enough to induce OM. Acetic acid, or with the addition of 5-FU, produces OM.	MDA contribute to oxidative stress, and higher carbamoyl-proxyl has been reported to be associated

			60mg/kg 5-FU<br>*N* = 8-11	Decay rate constant by probing for carbamoyl-proxyl	Acetic acid alone or with 5-FU had increased MDA concentration compared to saline and 5-FU controls ($p < 0.05$).<br><br>Decay of ROS was lower in 5-FU and acetic treatments, but higher in the acetic acid control group ($p < 0.05$).	with increased oxidative stress.
Vilar et al., 2020 [37]	Male golden Syrian hamsters.	Oral mucositis induced by 5-FU, and administration of gold nanoparticles (AuNP) with polyvinylpyrrolidone (PVP)<br>*N* = 5	Oral mucositis induced by 5-FU and administration of PVP<br>*N* = 5	IL-1β and GSH level quantified by ELISA assay<br><br>Histological analysis of oral tissues	AuNP treatment has reduced IL-1β and increased GSH level ($p < 0.0001$) and reduced oral mucositis symptoms following 5-FU pre-treatment ($p < 0.001$)	Increased GSH and reduced IL-1β associated with reduction of OM presentation
Gümüs et al., 2020 [38]	Twenty-one – thirty-day old female Wistar albino rats	Triamcinolone acetonide (TA) topical application (1.1% mg/gr) with 5-FU therapy (60mg/kg) intraperitonially OR chlorhexidine (CHX) oral rinse application (0.2 w/v) with 5-FU therapy (60mg/kg).<br>*N* = 10 each	Placebo group with NaCl oral rinse, Control group with 5-FU therapy (60mg/kg).<br>*N* = 8 each	GSH and LPO level measured to evaluate the preventive effect of TA and CHX on 5-FU induced oral mucositis.	GSH level was significantly higher in TA and CHX treatment groups compared to the control ($p < 0.001$).<br><br>LPO was reduced in both treatment groups ($p < 0.05$)	Greater GSH associated with reduced oral mucositis severity. Reverse is true for LPO.

Supplementary Table S3. Summary of *in vivo* study design and results of rebamipide as an intervention.

AUTHOR, YEAR	POPULATION	INTERVENTION	COMPARATOR	OUTCOME	EFFECT OBSERVED	MECHANISM OF ACTION
Takeuchi et al., 2018 [39]	Ten-week-old ICR mouse model of	Rebamipide-loaded PLGA nanoparticles coated with chitosan	Untreated, rebamipide	Ulceration area and duration of therapy	Reduction in ulcer area at days 9 ($p < 0.01$), 11 ($p < 0.01$) and 13 ($p < 0.01$) of rebamipide-loaded PLGA nanoparticle	No antioxidant mechanism assessed

	chemotherapy-induced OM	$N = 5$	suspension only or nanoparticles only $N = 5$ each		treatment compared to non-treated group.	
Nakashima et al., 2014 [40]	Rat model of radiotherapy-induced OM	2% rebamipide liquid with micro-crystals or submicronized crystals $N = 3\text{-}8$ per group	Purified water $N = 3\text{-}8$	Reduction ratio of rat oral ulcer<br><br>Ulcer-like percentage area of tongue	2% rebamipide liquid with submicronized crystals were more potent than micro-crystals in reducing radiation-induced oral ulcers and was dosage-dependent ($p < 0.01$).<br><br>Ulcer-like percentage area was lower in mice treated with rebamipide ($p < 0.05$).	No antioxidant mechanism assessed
Nakashima et al., 2017 [41]	Six-week-old Crl:CD Sprague-Dawley rat model of radiotherapy-induced OM	1, 2 or 4% rebamipide liquid $N = 10\text{-}12$ per group	Vehicle control $N = 11$	OM evaluation as damaged oral mucosa relative to area of tongue<br><br>Inflammatory gene and protein levels	Rebamipide had pre-treatment period-dependent ($p < 0.05$), dosing frequency-dependent ($p < 0.05$) and dose-dependent effect ($p < 0.01$) on reduction of tongue damaged area percentage.<br><br>Rebamipide had dose-dependent effect on reduction of inflammatory protein level and gene expression ($p < 0.01$).	No antioxidant mechanism assessed

Supplementary Table S4. Summary of *in vivo* study design and results of botanical extracts and natural compounds as interventions.

AUTHOR, YEAR	POPULATION	INTERVENTION	COMPARATOR	OUTCOME	EFFECT OBSERVED	MECHANISM OF ACTION
Kim et al., 2017 [25]	Seven-week-old golden hamster model of chemotherapy-induced OM	100, 500 or 1000μg/mL S. miltiorrhiza $N = 6$ each	Untreated $N = 6$<br><br>Naïve $N = 6$<br><br>Benzydimine HCl $N = 6$	Histological assessment of buccal mucosa<br><br>IL-1β, TNF-α, NF-κB and caspase-3 expression by western blot	S. miltiorrhiza treatment showed healed ulcerated oral mucosa.<br><br>Active caspase-3 expression was reduced in 500 and 1000μg/mL S. miltiorrhiza treated groups compared to control ($p < 0.05$ and < 0.01, respectively).<br><br>1000μg/mL S. miltiorrhiza also saw reduced TNF-α and IL-1β compared to the control (both $p < 0.05$).	Antioxidant or oxidative stress not assessed *in vivo*, but *in vitro.*

					Both 500 and 1000μg/mL S. miltiorrhiza saw increased cytoplasm NF-κB expression (both $p < 0.05$), but only 1000μg/mL treatment group saw lower nuclear NF-κB expression compared to the control ($p < 0.05$).	
Park et al., 2018 [26]	Seven-week-old male golden Syrian hamsters	Administration with onchung-eum (100, 500, or 1000 mg /kg) with the 5-FU treatment. *N* = 6 each	Only 5-FU treatment. *N* = 6 each	Cell viability<br><br>ROS production	Onchung-eum treatment led to significant recovery of the epithelial layers of oral mucosa after induction of chemotherapy-induced oral mucositis. Moreover, ROS production was significantly decreased in the treatment groups with onchung-eum compared to the control group with just 5-FU treatment ($p < 0.01$).	Reduction of ROS production contributed to improvement of OM presentation
Koohi-Hosseinabadi et al., 2015 [42]	Six – eight-week-old male golden hamsters model of chemotherapy-induced OM	*Z. jujuba*hydroalcoholic extract in topical gel (20%) or dietary form (300 mg/kg). *N* = 14 each	No treatment following 5-FU chemotherapy. *N* = 14	OM intensity associated with antioxidative stress protection as evaluated by SOD and MDA level	Both the topical and dietary application of Z. jujuba hydroalcoholic extract could reduce the intensity of chemotherapy-induced oral mucositis ($p < 0.05$), and the concentration of MDA, while increasing the activity of SOD ($p < 0.001$).	Not reported
Koohi-Hosseinabadi et al., 2017 [43]	Adult male golden hamster model of chemotherapy-induced OM	*E. angustifolia* hydroalcoholic extract in topical gel (10%) or dietary form (300 mg/kg). *N* = 14 each	No treatment following 5-FU chemotherapy. *N* = 14	The SOD level, MDA and MPO levels were measured to investigate the protective effect of E. angustifolia hydroalcoholic extract on chemotherapy-induced oral mucositis.	Both forms of E. angustifolia hydroalcoholic extract reduced MDA and MPO levels, while significantly increasing the SOD activity ($p < 0.05$).	SOD, MDA and MPO implicated in antioxidative stress protection in chemotherapy-model of OM.

Tanideh et al., 2019 [44]	Six-week-old golden male hamster model of radiotherapy-induced OM	1% T. ammi extract gel *N* = 18<br><br>3% P. atlantica extract gel *N* = 18<br><br>1% T. ammi extract + 3% P. atlantica extract gel *N* = 18	Untreated *N* = 18	Volume density of epithelium by stereology assessment<br><br>Tissue MDA level, MPO and SOD activity<br><br>Tissue IL-1β level	P. atlantica and T. ammi either alone or together, after 13 days of treatment, had increased oral epithelium density ($p < 0.01$, each).<br><br>Tissue MDA, MPO and IL-1β levels were reduced in P. atlantica, T. ammi and as a mixed gel at days 13 ($p < 0.001$ for P. atlantica; p<0.01 for T. ammi), 15 (both $p < 0.001$) and 17 (both $p < 0.001$). Mixed treatment was reduced for days 13, 15 and 17 (all $p < 0.001$). Furthermore, mixed treatment was more effective than either treatment alone ($p < 0.01$).<br><br>SOD activity was higher in the three treatment types compared to untreated ($p < 0.05$). P. atlantica was more effective than T. ammi for SOD activity on days 15 ($p < 0.001$) and 17 ($p = 0.004$).	Higher MDA and MPO are associated with greater oxidative stress.<br><br>SOD negatively associated with oxidative stress.
Watanabe et al., 2013 [45]	Seven-week-old golden hamster model of chemotherapy-induced OM	10 or 30% royal jelly contained in chitosan-sodium alginate film *N* = 6-12 each	Naïve *N* = 6<br><br>Film *N* = 12<br><br>Untreated *N* = 6-12	Ulcer area<br><br>MPO activity expressed as unit/g cheek pouch<br><br>IL-1β and TNF-α expression via western blot analysis<br><br>Histopathological evaluation	10 and 30% royal jelly film, compared to film and untreated groups, reduced the area of OM ($p < 0.05$).<br><br>MPO activity that had increased in the control group, following chemical insult, was reduced in the royal jelly groups ($p < 0.05$).<br><br>IL-1β and TNF-α expressions were also increased in the untreated group but reduced in the royal jelly groups ($p < 0.01$; 30% RJ v control for TNF-α $p < 0.05$).	MPO associated with greater oxidative stress.

					There was fewer infiltration of inflammatory cells, reduced ulceration and abscesses in the royal jelly treated groups. Epithelial healing was observed in this group.	
Takuma et al., 2008 [46]	Male Syrian golden hamsters of six weeks old.	Administration of *E. japonica* seed extract (10ml/day) after oral mucositis was induced by 5-FU treatment. *N* = 6 each	Control group had Administration of water instead of E. japonica seed extract after 5-FU treatment. Normal group had no treatment. *N* = 6	OM features evaluated graded<br><br>Plasma lipid peroxide level quantified	Reduced mucositis score in treated group compared to negative controls ($p < 0.05$).<br><br>Lipid peroxide level comparable to naïve group but reduced compared to negative controls ($p < 0.05$).	Reduction in lipid peroxide associated with lower mucositis score
Rezvani & Ross, 2004 [47]	Twelve-week-old Sprague-Dawley rat model of radiotherapy-induced OM	0.5mL compound A *N* = 8	Untreated *N* = 8<br><br>Sunflower oil *N* = 8<br><br>α-tocopherol *N* = 8	Oral mucositis incidence in irradiated rats	Sunflower oil and α-tocopherol required more radiation to induce OM in rats; each require 1.05 and 1.09 times more than untreated mice, respectively. Compound A required 1.2 times more radiation for onset of OM ($p < 0.001$).	No assessment of antioxidation. Sunflower oil and α-tocopherol have reported for antioxidative effect.
Gupta et al., 2020 [48]	Ten-week-old female C3H mice	Intraperitoneal injection of either PBS or 5-FU with L. reuteri containing water *N* = 12 each	Intraperitoneal injection of either PBS or 5-FU with water *N* = 12 each	Expression of oxidative stress markers and antioxidant genes<br><br>Histological alterations of the oral mucosal tissues	In the experiment groups, epithelial damage was significantly lower compared to control groups ($p < 0.001$) Moreover, oxidative stress was reduced in the oral mucosal tissues ($p < 0.001$).	Not reported
Cuba et al., 2020 [49]	Ten-week-old male CF-1 mouse model of	3, 10 or 30mg/kg cannabidiol *N* = 5-9 each	Placebo *N* = 9 each	OM evaluation<br><br>Histological analysis of inflammatory response	Reduced OM grade with 7 days of 30mg/kg cannabidiol ($p < 0.01$), but reduced inflammation only for 3 and 10mg/kg cannabidiol.	Catalase and glutathione associated with reduced OM grade.

	chemotherapy-induced OM			Glutathione levels and catalase activity by biochemical analysis	Glutathione levels were reduced in mice treated with cannabidiol for 7 days compared to 4 days ($p < 0.01$).<br><br>Catalase activity reduced in 7-day treatment compared to 4-day treatment ($p < 0.01$).	
Aghel et al., 2014 [50]	Seven – eleven-week-old Wistar rat model of radiotherapy-induced OM	400mg/kg propolis *N* = 7	Saline *N* = 7	OM score<br><br>Antioxidant evaluation	Mucositis severity was higher in saline group than propolis treated group ($p = 0.025$).<br><br>Salivary antioxidants higher in propolis treated group at day 0, and lowered after 10 days of daily propolis treatment ($p = 0.041$)	Greater, but statistically insignificant different, salivary antioxidants associated with less mucositis severity. However, no specific antioxidant mechanism
Motallebnejad et al., 2020 [51]	Ten – twelve-week-old Wistar albino rat model of radiotherapy-induced OM	25 or 50mg/kg lycopene *N* = 7 each	50mg/kg lycopene without irradiation *N* = 7<br><br>Vehicle control *N* = 7<br><br>Time control *N* = 7	Mean weight<br><br>OM grade<br><br>Antioxidant activity by FRAP assay<br><br>Lipid peroxidation level by TBARS assay	Mean weight not affected by treatment type but reduced after 10 days for all groups ($p < 0.001$).<br><br>Only 50mg/kg lycopene treatment after irradiation was able to prevent OM grade of more than 2. Both lycopene concentrations scored lower for OM grade on day 3 (25mg/kg: $p < 0.05$; 50mg/kg: $p < 0.001$) and on day 6 (25mg/kg: $p < 0.05$; 50mg/kg: $p < 0.001$) post-irradiation. On day 6, 50mg/kg lycopene reduced the OM grade further than 25mg/kg ($p < 0.001$).<br><br>FRAP assay reading was higher for all tested concentrations of lycopene regardless of irradiation (50mg/kg without irradiation: $p < 0.001$; 25mg/kg: *p*	Antioxidant activity was higher after 50mg/kg lycopene treatment. Lipid peroxidation was lowered in lycopene treatment to reduce oxidative stress.

					< 0.05; 50mg/kg: $p < 0.001$) and TBARS assay reading was elevated in the irradiated control ($p < 0.001$), and irradiated lycopene groups (both $p < 0.05$) at day 10. TBARS levels was reduced in 25 and 50mg/kg lycopene with irradiation ($p < 0.05$ and $p < 0.001$, respectively).	

Supplementary Table S5. Summary of *in vivo* study design and results of MnBuOE as an intervention.

AUTHOR, YEAR	POPULATION	INTERVENTION	COMPARATOR	OUTCOME	EFFECT OBSERVED	MECHANISM OF ACTION
Birer et al., 2017 [52]	Six – eight-week-old C56BL/6 female mice	Subcutaneous MnBuOE (a redox-active manganese-porphyrin dissolved in saline) injection, along with radiotherapy. *N* = 8-13 each	Saline administration along with radiotherapy. *N* = 8-13 each	GSH/GSSG ratio was measured to evaluate the protective effect of MnBuOE against the radiotherapy-induced oral mucositis.	In the control group, radiotherapy without any additional treatment led to decrease in GSH/GSSG ratio indicating increase in the oxidative stress. In the experimental group, addition of MnBuOE led to increase in GSH/GSSG ratio compared to the control group.	GSH/GSSG increase associated with radiotherapy-induced OM protection
Ashcraft et al., 2015 [53]	C57Bl/6 mice	Subcutaneous MnBuOE injection with irradiation targeted to salivary glands and oral cavity. *N* = 3 each	No irradiation. *N* = 3 each	Protective effect of MnBuOE against radiotherapy-induced oral mucositis.	MnBuOE led to significant reduction in radiotherapy-induced oral mucositis (p = 0.009). Overall, MnBuOE can widen the therapeutic margin of radiation dose, which gives more power to control tumours.	Not reported

Supplementary Table S6. Summary of *in vivo* study design and results of photobiomodulation as an intervention.

AUTHOR, YEAR	POPULATION	INTERVENTION	COMPARATOR	OUTCOME	EFFECT OBSERVED	MECHANISM OF ACTION
Cruz et al., 2015 [54]	Female Syrian golden hamsters.	Group (aPDT)received antimicrobial laser and photodynamnic therapy (aPDT) along with 5-FU chemotherapy. *N* = 54	Group (C) with just vehicle of chemotherapy, Group (Ch) received just 5-FU chemotherapy *N* = 36<br><br>Group (ChP) received methylene blue (0.01%) along with 5-FU *N* = 48<br><br>Group (ChL) received methylene blue (0.01%), LDL irradiation, and 5-FU treatment. *N* = 55	SOD and catalase levels were measured to evaluate the protective effect of aPDT against the chemotherapy-induced oral mucositis.	The experimental group with aPDT treatment led to most healing of OM (76%), and the persistence of the oral mucositis was the lowest for this group compared to control and any other experimental groups (p = 0.67172). SOD was increased ($p < 0.05$), and catalase ($p < 0.05$) decreased after seven days	Increased SOD and reduced catalase were associated with OM healing
Thieme et al., 2020 [55]	Rat model of chemotherapy-induced OM	Extra-oral 6 J/cm^{2}laser irradiation *N* = 18<br><br>Extra-oral 12 J/cm^{2}laser irradiation	Naïve *N* = 6<br><br>Untreated *N* = 18	OM scoring<br><br>Histological score<br><br>Epithelial nitrotyrosine score	OM score only reduced in extra-oral 6 J/cm^2 laser irradiation at day 8 compared to untreated group ($p < 0.05$). Other modes of laser irradiation reduced on days 10 and 14 ($p < 0.05$).<br><br>Histologically there was improved epithelial repair and reduced inflammation in OM in	Glutathione peroxidase increased by photobiomodulation. This provides better neutralisation of ROS.

		$N = 18$<br><br>Intra-oral 6 J/cm^2 laser irradiation<br>$N = 18$		DCFH oxidation level<br><br>Glutathione peroxidase, catalase and glutathione activity	groups of all laser irradiation modalities on day 8 ($p < 0.05$).<br><br>Laser irradiation modalities similar had similar response to epithelial nitrotyrosine, catalase or glutathione levels compared to untreated mice.<br><br>DCFH oxidation was reduced in the extra-oral 6 J/cm^2 laser irradiation group on day 8 compared to untreated mice ($p < 0.05$).<br><br>Glutathione peroxidase activity was significantly increased for all modalities of laser irradiation only on day 8 ($p < 0.05$). No difference between groups for catalase or glutathione activity	

Supplementary Table S7. Summary of *in vivo* study design and results of transplant as an interventions.

AUTHOR, YEAR	POPULATION	INTERVENTION	COMPARATOR	OUTCOME	EFFECT OBSERVED	MECHANISM OF ACTION
Shen et al., 2018 [56]	Male mice (C57)	*In vivo* transplantation of CXCR2-overexpressing MSCs.<br>$N = 6$	No treatment.<br>$N = 6$	ROS level measured to see protective effect of CXCR2-overexpressing MSCs transplantation against radiotherapy-induced oral mucositis.	Treatment led to significant reduction in ROS production ($p < 0.05$) and accelerated healing of radiotherapy-induced oral mucositis.	Increased CXCR2 expression associated with reduction of ROS, inflammatory cytokines and improved OM healing

Supplementary Table S8. Summary of *in vivo* study design and results of antioxidants.

AUTHOR, YEAR	POPULATION	INTERVENTION	COMPARATOR	OUTCOME	EFFECT OBSERVED	MECHANISM OF ACTION
Greenberger et al., 2014 [7]	Fanconi anaemia (Fancd2−/−) mice of six to eight weeks old.	GS-nitroxide therapy (JP4-039), along with irradiation to induce oral mucositis. *N* = Not reported	Control group received F15 emulsion alone. *N* = Not reported	DNA double stranded break was measured, and histopathologic evaluation of oral mucosa was conducted to evaluate the protective effect of GS-nitroxide therapy (JP4-039) against radiation-induced oral mucositis.	GS-nitroxide therapy allowed significant reduction in radiation-induced oral mucositis ($p < 0.05$).	GS-nitroxide reported for antioxidant properties but no mechanism of the oxidative stress pathway was evaluated
Shinde et al., 2016 [24]	Ten – twelve-week-old Fancd2+/+, Fancd2+/− and Fancd2−/− C57BL/6 mice	Radiotherapy (single 28 Gy or fractioned dose 8 Gy x 4) and administration of F15/JP4-039 nitroxide OR F15/4-amino-Tempo *N* = 3-4 each	Radiotherapy (single 28 Gy or fractioned dose 8 Gy x 4) and administration of F15 only. *N* = 3-4 each	Histological evaluation to investigate the protective effect of JP4-039 and 4-amino-Tempo against radiotherapy-induced oral mucositis.	Only JP4-039 treatment led to improvement of oral mucositis ($p < 0.0001$).	Not reported
Willis et al., 2018 [57]	Double-knockout [SMAD3−/− (129/Sv)/Fancd2−/−(129	Mitochondrial-targeted GS-	F15 alone *N* = 20	OM severity by ulcer per oral mucosa area	Reduced OM affected area percentage ($p < 0.05$)	No mechanism of oxidative stress pathway

	/Sv)] mice, Fancg−/−, Fanca−/−, Fancd2−/−	nitroxide, JP4-039/F15<br>*N* = 20		via histopathological analysis<br><br>Western blot analysis of TGF-β pathway<br><br>RT-PCR for TGF-β mRNA expression	Reduced irradiation-mediated TGF-β mRNA elevation and plasma protein ($p < 0.05$).	examined, but GS-nitroxide is a known antioxidant.
Cortim et al., 2012 [58]	Rat model of radiotherapy-induced and chemoradiotherapy-induced OM	137.5 or 275mg/kg tempol<br>*N* = 8 per group<br><br>150mg/kg D-met only<br>*N* = 4-8	No irradiation and/or 5-FU<br>*N* = 4-8<br><br>Untreated<br>*N* = 4-8<br><br>L-met only<br>*N* = 4	Mean ulcer size<br><br>Mean tongue epithelial thickness by histological analysis	275mg/kg tempol reduced the fractionated dose radiation-induced ulcer size ($p < 0.001$) and had higher mean epithelial thickness at 7- and 8-day post-irradiation compared to untreated ($p < 0.001$, each).<br><br>Chemoradiotherapy-induced ulcers were reduced in size in 137.5 and 275mg/kg tempol treatments compared to untreated groups ($p < 0.001$).<br><br>D-met group showed reduced ulcer size compared to the untreated group ($p < 0.001$). D-met groups had lower, but statistically insignificant ($p > 0.05$), ulcer size in chemoradiotherapy mouse models, compared to the untreated group.	Not assessed, but tempol is an antioxidant.
Hu et al., 2017 [59]	Miniature pig model of radiotherapy-induced OM	30mg/kg tempol<br>*N* = 4	Naïve<br>*N* = 4	Oral mucositis score<br><br>Percent area of ulcer	30mg/kg tempol treated groups had lower oral mucositis scores at day 15	Not assessed, but tempol is an antioxidant.

			Untreated<br>$N = 4$	Oral epithelium thickness by histological analysis	and 18 ($p < 0.05$) and had lower percent area of ulcers on the tongue ($p < 0.05$).<br><br>Only the buccal epithelial thickness was higher in the tempol treated minipigs compared to the untreated group – the thickness was equivalent to the naïve group.	
Üçüncü et al., 2006 [60]	Rat model of radiotherapy-induced OM	Vitamin E<br>$N = 7$<br><br>L-carnitine<br>$N = 7$<br><br>Mix<br>$N = 7$	Naïve<br>$N = 7$<br><br>Untreated<br>$N = 7$	Evaluation of OM severity following Parkins et al scale<br><br>Histopathological assessment of oral epithelium<br><br>Assessment of MDA, SOD and CAT level from blood samples	Vitamin E and L-carnitine groups had a later onset of OM than untreated groups ($p < 0.05$).<br><br>Histologically, vitamin E and L-carnitine treated rats had fewer inflammatory infiltration, vacuolar alteration of basal layer and epithelial cellular changes ($p < 0.05$).<br><br>MDA level had decreased, and SOD and CAT enzymes increased in the plasma when treated with vitamin E or L-carnitine ($p < 0.05$). The same was observed with the mixed treatment ($p < 0.05$); however, the decrease for MDA was statistically insignificant ($p > 0.05$).	MDA decrease, and SOD and CAT increase in the blood plasma was associated with reduce inflammatory infiltration and delayed OM onset

Supplementary Table S9. Summary of *in vivo* study design and results of gene therapy as an intervention.

AUTHOR, YEAR	POPULATION	INTERVENTION	COMPARATOR	OUTCOME	EFFECT OBSERVED	MECHANISM OF ACTION
Luo et al., 2019 [61]	Radiotherapy-induced OM female mice model with human oral tissue xenotransplant	Tat-Smad7<br>*N* = Not reported	30% glycerol in PBS vehicle control<br>*N* = Not reported<br><br>Naïve<br>*N* = Not reported	Tumour area<br><br>Ulcer size<br><br>DNA damage assessed by pH2AX and 8-OHdG<br><br>Cell apoptosis by TUNEL labelling<br><br>Leukocyte infiltration by F4/80 immunostaining<br><br>Cell proliferation	Tumour size reduced due to radiotherapy alone and with Tat-Smad7 (both $p < 0.05$).<br><br>Ulcer size was reduced by Tat-Smad7 treatment as well ($p < 0.05$).<br><br>Tat-Smad7 treated mice had fewer pH2AX and 8-OHdG (both $p < 0.05$).<br><br>Tat-Smad7 treated mice had fewer TUNEL labelled cells ($p < 0.05$).<br><br>Tat-Smad7 treated mice had unchanged pH2AX expression (p>0.05) but increase 8-OHdG expression ($p < 0.05$).<br><br>Intervention group had reduced F4/80+ cell density compared to vehicle control ($p < 0.01$).<br><br>The intervention group oral mucosa saw more BrdU stained cells, by density counts, compared to the control ($p < 0.01$).	8-OHdG, a marker of oxidative DNA damage due to ROS, was reduced and was associated with smaller ulcerative lesions following radiotherapy and Tat-Smad7 treatment
Guo et al., 2003 [62]	C3H/HeNsd mice	Plasmid/liposome complexes with Plasmid DNA of human SOD2 transgene or *LacZ* transgene.	Control mice: no treatment of plasmid DNA of human SOD2 transgene- just saline.	SOD2 level was measured to evaluate the protective effect of SOD2 against	SOD2 plasmid complex treatment led to significant increase in SOD2 level ($p = 0.029$), and significantly decreased amount of ulceration present compared to the group that received *LacZ*-transgene ($p = 0.04$).	Increased SOD2 expression associated with reduced

		N = 5-12 each	*N* = 12	radiation-induced oral mucositis.		OM-associated ulceration
Epperly et al., 2004 [63]	Rat model of radiotherapy-induced OM	Manganese superoxide dismutase-plasmid/liposome *N* = 5	Amifostine *N* = 5<br><br>No treatment *N* = 5	Histological analysis of OM tongue tissue<br><br>Mucosal cell cycling by BuDR labelling<br><br>Saliva output<br><br>Oral mucosal cell apoptosis	Percentage of sections with ulcerated oral epithelium were reduced in MnSOD-PL treated group ($p < 0.05$).<br><br>No statistically significant influence on cell cycling by MnSOD-PL treatment.<br><br>Saliva output reduction was mitigated with MnSOD-PL ($p < 0.05$).<br><br>Percentage of apoptotic cells were significantly reduced in rats treated with MnSOD-PL ($p \leq 0.0001$).	MnSOD plasmid treatment associated with reduction of apoptotic cell death and OM-mediated ulceration

Supplementary Table S10. Summary of *in vivo* study design and results of irradiation as an intervention.

AUTHOR, YEAR	POPULATION	INTERVENTION	COMPARATOR	OUTCOME	EFFECT OBSERVED	MECHANISM OF ACTION
Tao et al., 2019 [64]	Mouse model of radiotherapy-induced OM	3, 5 and 7 days after 25Gy irradiation *N* = 5 each	No irradiation *N* = 5 each	miR-200 expressions<br><br>Proinflammatory cytokine mRNA expressions	miR-200a ($p < 0.001$), -200b ($p < 0.001$) and -200c ($p < 0.05$) were increased after 4 days irradiation compared to untreated mice. These return to levels comparable to non-irradiated group.<br><br>TNF-α, MIP-1β and IL-1α expression after 7 days of irradiation compared to non-irradiated ($p < 0.0001$). TGF-β and IL-6 showed peak expression at days 5 ($p < 0.001$) and 7 ($p < 0.0001$) after irradiation, respectively.	miR-200 reported in the study to regulate ROS production, covered in *in vitro* experiments.

Clinical Studies

Supplementary Table S11. Summary of results of Vitamin E as an intervention.

AUTHOR, YEAR	STUDY TYPE	POPULATION	INTERVENTION	COMPARATOR	OUTCOME	EFFECT OBSERVED	MECHANISM OF ACTION
Sung et al. 2007 [65]	Series of N-of-1 double-blinded RCTs	Children between 6-17 years old undergoing CT *N* = 53	Vitamin E 800mg *N* = 22	Placebo *N* = 23	OM assessed according to scale developed by Sonis et al.	No significant difference in objective mucositis scores between the intervention group (0.2) and the control group (0.3).	Antioxidant activity
Ferreira et al., 2004 [66]	Double-blind RCT	Patients with cancer of the oral cavity and oropharynx *N* = 54	Vitamin E (alpha-tocopherol, 400mg) *N* = 28	500mg placebo *N* = 26	OM via toxicity criteria of the Radiation Therapy Oncology Group and the European Organization for Research and Treatment of Cancer (RTOG/EORTC) objective grading system	Symptomatic OM more frequent in the placebo group than in the VE group (p = 0.038) Subjective pain scores indicated VE reduced pain grades during RT (p = 0.0001).	Vitamin E has antioxidant activity.
El-Housseiny et al., 2007 [67]	RCT	Paediatric patients undergoing chemotherapy with OM *N* = 80	Vitamin E topically b.i.d. (100mg) *N* = 40	Vitamin E systemically *N* = 40	OM via WHO oral toxicity scale	80% of treated with VE topically had complete resolution of OM after 5 days, whereas 93.9% of those in the systemic administration group had failed to improve in OM grade.	
Wadleigh et al., 1992 [68]	Double-blind RCT	Patients receiving chemotherapy to treat various malignancies *N* = 18	topical vitamin E oil (1ml, 400mg/mL), on oral lesions, b.i.d *N* = 9	Placebo (coconut + soybean oil) *N* = 9	OM via WHO oral toxicity scale	6 out of 9 of the VE group had full recovery of OM, whereas the control group had 8 out of 9 subjects did not recover, difference statistically significant (p = 0.025).	

Pentoxifylline + Vit E							
Sayed et al., 2019 [69]	Prospective RCT	H&N cancer patients receiving 30-35 RT fractions with or without CT were included $N = 60$	Pentoxifylline and vitamin E $N = 30$	No pentoxifylline nor vit E $N = 30$	Severity of oral symptoms graded using national cancer institute common terminology criteria for adverse events version 4.03.	Pentoxifylline and vitamin E did not affect the incidence of OM, however, after adjusting for age, it decreased the incidence of severe OM (p = 0.01) and decreased the duration of OM by 5 weeks (p = 0.002).	Pentoxifylline is non-specific phosphodiesterase inhibitor that blocks transcription of TGF-β1; Vit E is an antioxidant that inhibits production of TGF-β1.
Azelastine (2 mg) + vitamin C (500 mg) + vitamin E (200) + glutathione (200 mg)							
Osaki et al., 1994 [6]	Controlled Clinical Trial	Patients receiving chemotherapy $N = 63$	Azelastine (2mg), vitamin C 500mg, vitamin E 200mg and glutathione 200mg $N = 37$	Control: vitamin C 500mg, vitamin E 200mg and glutathione 200mg $N = 26$	OM graded based on erythema, severity of ulceration and effect on patient diet	Inflammation occurred later and less severely in the Azelastine group. (p not reported)<br>The control group required longer for Grade 4 OM to resolve. ($p < 0.05$)<br>O_2- generation was lower in the Azelastine group (p < 0.05), no correlation was found between O_2- levels and severity of OM.	Azelastine stabilises cell membrane, has capacity to suppress leukocytes. vitamin C and vitamin E have antiradical activities. Glutathione reduces oxidants.
Treatment combining Vit E, triamcinolone and hyaluronic acid							
Agha-Hosseini et al., 2021 [70]	Triple-blind RCT	Patients with H&N cancer undergoing RT on an outpatient basis $N = 60$	Mouthwash containing Vitamin E 0.2%, Triamcinolone 0.1%, and	Triamcinolone mouthwash 0.1% $N = 30$	Severity of OM determined by WHO classification and pain intensity measured by numerical pain intensity scale	Significant higher reduction in OM grade ($p < 0.001$) and pain intensity ($p < 0.001$) in the intervention group.	Antioxidant, anti-inflammatory, and improved healing process mechanisms.

			Hyaluronic acid 0.2% *N* = 29				
				Vitamin E vs Pycnogenol (Pine bark extract)			
Khurana et al., 2013 [71]	Single Blind RCT	Children with chemotherapy induced OM *N* = 72	Group II: Vitamin E (200mg) (n=24) Group III: Pycnogenol (1mg/kg) *N* = 24	Group I: Control (Glycerine) *N* = 24	OM via WHO oral toxicity scale, Oral Mucositis Assessment Scale (OMAS), and Children's International Mucositis Evaluation Scale (ChIMES)	Statistically, both intervention arms were equivalent in effect - both treatments reduced severity of OM and pain was reduced regardless of treatment modality. ($p = 0.998$)	Both interventions are strong antioxidants.

Supplementary Table S12. Summary of results of Genetic Influences and Inherent Antioxidants.

AUTHOR, YEAR	STUDY TYPE	POPULATION	INTERVENTION	COMPARATOR	OUTCOME	EFFECT OBSERVED	MECHANISM OF ACTION
Venkatesh et al., 2014 [72]	Observational	H&N cancer patients undergoing chemo-radiotherapy or RT *N* = 183	Post chemo/RT	Prior to chemo/RT	Degree of OM assessed according to RTOG and genetic polymorphism via genomic DNA isolation	NBN variants are of predictive significance ($p = 0.023$) in analysing oral mucositis prior to radiotherapy.	SNPs in specific genes relate to DNA damage and repair, serve as a biomarker for predicting tissue toxicity.
Pratesi et al., 2011 [73]	Observational Study	Patients with squamous cell carcinoma of the head and neck (SCCHN) after radiotherapy (RT) *N* = 101			Genetic polymorphisms detected via high resolution melting analysis (HRMA) Radiation induced toxicities via Common	XRCC1-399Gln allele significantly associated with higher risk of OM ($p = 0.011$) Development of grade ≥2 OM was increased in	Genes tested are protective against oxidative stress.

					Toxicity Criteria for Adverse Events (CTCAE)	patients with the XRCC1-399G1n allele. ($p = 0.001$) The presence of one or more SNP in XRCC1 p.G1n399Arg/RAD51 c.-3429 G > C was associated with a higher likelihood of acute toxicities. ($p = 0.004$)	
Urbain et al., 2012 [74]	Prospective study	Adults (haematological malignancies) treated with allogenic haematopoietic cell transfer (alloHCT) with chemotherapy $N = 70$			Severity of OM via WHO oral toxicity scale AOX status in buccal mucosa cells (BMC) and plasma	There was no significant difference found in baseline AOX concentrations between the patients with various severities of OM. (*p* value not reported) Tendency for patients with sub-normal AOX levels to require a longer duration of parenteral nutrition. ($p = 0.066$) No single AOX had predictive value for incidence or severity of OM. (*p* value not reported)	
Wardman et al., 2013 [75]	Observational Study	Patients with carcinomas of the head and neck treated with CHART (radiotherapy) $N = 18$			Glutathione, cysteine, urate, and ascorbate measured in plasma. OM distribution	There was no correlation found between mucositis severity and measures of plasma AOX. ($p = 0.12$)	
Bachmeier et al., 2014 [76]	Longitudinal observational study	Bone marrow transplant patients $N = 20$	Post BMT	Prior to BMT	Superoxide dismutase and Uric acid activity using RANDOX and UOD/ PAP	Post BMT, 85% developed OM, increase in SOD ($p < 0.01$) and	SOD regarded as OM defence mechanism; UA

					spectrophotometry, respectively. OM using WHO classification	decrease in UA ($p < 0.001$) during M-stage.	level reflects progression of OM.
Severin et al., 2005 [77]	Human in vitro and in vivo	Leukemia patients receiving BMT (*N* = 40) and healthy blood donors (*N* = 67)	Post-radiation	Pre-radiation	Leukocytes of CD34+ HBSC, lymphocytes and plasma antioxidant concentration using ABTS and FRAP assay	Severe OM associated with specific depletion in leukocyte ($p = 0.008$), lymphocyte ($p = 0.008$) and plasma antioxidant concentration ($p < 0.001$).	

Supplementary Table S13. Summary of results of rebamipide as an intervention.

AUTHOR, YEAR	STUDY TYPE	POPULATION	INTERVENTION	COMPARATOR	OUTCOME	EFFECT OBSERVED	MECHANISM OF ACTION
Chaitanya et al., 2017 [78]	Double blind RCT	Chemo-radiotherapy patients *N* = 60	Rebamipide gargle 100mg/day *N* = 30	Placebo gargle *N* = 30	Degree of OM assessed subjectively by Numeric rating scale and objectively via RTOG grading	Rebamipide led to decreased severity ($p = 0.001$) and pain intensity ($p = 0.001$), delayed onset ($p = 0.012$).	Antioxidant properties
Ishii, 2017 [79]	Non-controlle d trial	Patients with stomatitis who underwent chemotherapy *N* = 5	Rebamipide mouthwash (25mL)		OM via National Cancer Institute's Common Terminology Criteria for Adverse Events (CTCAE)	3/5 patients' stomatitis evaluation score (SES) reached 0. Remaining patients experienced an improvement of SES score. (*p* value not reported)	Rebamipide inhibits ROS generation.

Supplementary Table S14. Summary of results of zinc/polaprezinc as an intervention.

AUTHOR, YEAR	STUDY TYPE	POPULATION	INTERVENTION	COMPARATOR	OUTCOME	EFFECT OBSERVED	MECHANISM OF ACTION
Gholizadeh et al., 2017 [80]	Double-blind RCT	Patients with acute myeloid leukemia undergoing CT *N* = 140	Zinc sulfate 220mg orally *N* = 70	Placebo capsule *N* = 70	OM severity according to WHO.	Frequency of severe OM reduced in treatment compared to control group ($p = 0.004$).	Antioxidant and protective agent
Doi et al., 2015 [81]	Prospective study	Newly diagnosed patients with H&N cancer and undergoing RT *N* = 32	Polaprezinc mouthwash 150mg/day *N* = 32	Past H&N cancer patients *N* = 30 who underwent RT without PZ rinse	Severity of OM using Common Terminology Criteria for Adverse Events, version 3	PZ group had decreased incidence of grade 3 OM ($p = 0.18$), PZ also promoted recovery.	Antioxidant properties and scavenges free radicals.

Supplementary Table S15. Summary of results of selenium as an intervention.

AUTHOR, YEAR	STUDY TYPE	POPULATION	INTERVENTION	COMPARATOR	OUTCOME	EFFECT OBSERVED	MECHANISM OF ACTION
Büntzel et al., 2010 [82]	Randomised observational studies	RT patients *N* = 121	Selenium 500ug RT days and 300ug on weekend *N* = 63	No selenium *N* = 64	Selenium serum concentration measured by Atom Absorption Spectrometry.	Decrease of selenium concentration at 50% and end of RT ($p < 0.0001$). At beginning and 6 weeks post radiation no significant differences between groups was observed.	Selenium is essential cofactor of the glutathione peroxidase which is important for endogenous detoxification of free radicals.
Jahangard-Rafsanjani et al., 2013 [83]	Double blind, RCT	Leukaemia patients undergoing hematopoietic stem cell transfer (HSCT) with high-dose chemotherapy (HDC) *N* = 77	Selenium 200mcg b.i.d. for 14 days *N* = 38	Placebo *N* = 39	OM incidence and grade via WHO oral toxicity scale, Glu.Px activity	Cumulative incidence and duration not significantly different between groups. (p = 0.76) Occurrence of Severe OM significantly reduced in Selenium group. ($p = 0.013$) Time taken to improve from grade 4 to grade 2 OM shorter in selenium group. ($p = 0.014$)	Selenium is a cofactor for Glu.Px, a free radical scavenger.

						Glu.Px ($p = 0.008$) and selenium ($p = 0.018$) elevated in serum of intervention.	

Supplementary Table S16. Summary of results of photobiomodulation as an intervention.

AUTHOR, YEAR	STUDY TYPE	POPULATION	INTERVENTION	COMPARATOR	OUTCOME	EFFECT OBSERVED	MECHANISM OF ACTION
Rupel et al., 2018 [20]	Human in vivo and in vitro	Patients affected by grade 2 or 3 OM aged between 40-95 years, diagnosed with solid or haematologic malignancy undergoing CT and/or RT, and available to undergo PBM for 4 consecutive days *N* = 10	Photobiomodulation	No PBM	Oxidative stress status measured in saliva using TOS method, evaluation of intracellular ROS production and kinetics in PMN, real-time quantification of oxidative stress, keratinocyte viability and oxidative stress assay following chemotherapy, gene expression analysis.	660nm laser light increases ROS, whereas the 970nm light exerted a moderate antioxidant activity ($p < 0.0001$). The 800nm light or the combination of the 3 wavelengths resulted in the largest ROS reduction ($p < 0.001$).	Red and near-infrared light at low irradiance excites cytochrome c oxidase which represents the main source of intracellular ROS generation in the mitochondria.

Supplementary Table S17. Summary of results of hyaluronic acid-based compound as an intervention.

AUTHOR, YEAR	STUDY TYPE	POPULATION	INTERVENTION	COMPARATOR	OUTCOME	EFFECT OBSERVED	MECHANISM OF ACTION

Cirillo et al., 2015 [19]	Case series	Patients receiving radio- and/or chemotherapy *N* = 5	Mucosamine	-	OM via visual analogue scale	Prevention or reduced grading of OM following Mucosamin spray application, thrice daily, for 4 days prior to every chemo- and/or radiotherapy cycle.	
Bardellini et al., 2016 [84]	Double-blind RCT	Paediatric patients receiving chemotherapy for acute lymphoblastic leukemia with OM grade 1 or 2 *N* = 56	Mucosyte *N* = 28	Placebo *N* = 28	OM via WHO oral toxicity scale Pain via Visual Analogue Scale	The intervention arm had a statistically significant decline of OM after 3 days ($p = 0.0038$), and a statistically significant difference in pain reduction compared to the control at 3 and 8 days ($p < 0.005$).	

Supplementary Table S18. Summary of results of Genistein (soy isoflavones) as an intervention.

AUTHOR, YEAR	STUDY TYPE	POPULATION	INTERVENTION	COMPARATOR	OUTCOME	EFFECT OBSERVED	MECHANISM OF ACTION
Tacyildiz et al., 2010 [85]	Pilot study	Children receiving chemo±radio-therapy *N* = 6	Genistein supplement (8mg) once daily	No treatment (cross-over design)	Duration of adverse effects (OM)	Patients experienced less OM when treated with genistein during chemotherapy. Serum levels of genistein during treatment were 2-6 times higher than prior to supplementation. (*p* value not reported)	Genistein has antioxidant capacity.

Supplementary Table S19. Summary of results of Actovegin as an intervention.

AUTHOR, YEAR	STUDY TYPE	POPULATION	INTERVENTION	COMPARATOR	OUTCOME	EFFECT OBSERVED	MECHANISM OF ACTION
Wu et al., 2010 [86]	RCT	Nasopharyngeal carcinoma patients receiving concomitant chemoradiotherapy ±	Actovegin (1200mg) as Prevention *N* = 53	Control (No Treatment) *N* = 51	OM via National Cancer Institute's Common Toxicity Criteria (NCI CTC 2.0)	Intervention arms had fewer incidences of grade 2 OM (73.6% and 35.9% compared to 92.3%). ($p = 0.023$, $p = 0.035$)	

		induction chemotherapy<br>*N* = 156	Treatment<br>*N* = 51		Pain via Verbal Rating Scale Criteria	Grade 3 OM incidence lower for the prevention arm (26.4%) compared to the control (55.8%). ($p = 0.002$)<br>Actovegin reduced patient grading of pain when used as a prevention, but not as therapy. ($p = 0.011$)	

Supplementary Table S20. Summary of results of GC4419 as an intervention.

AUTHOR, YEAR	STUDY TYPE	POPULATION	INTERVENTION	COMPARATOR	OUTCOME	EFFECT OBSERVED	MECHANISM OF ACTION
Anderson et al., 2019 [87]	Phase IIb, double-blind RCT	Patients with locally advanced oral or oropharyngeal cancer<br>*N* = 223	GC4419 30mg<br>*N* = 73<br><br>or 90mg<br>*N* = 76	No GC4419<br>*N* = 74	OM severity by WHO criteria	Dose of 30mg produced intermediate improvements, and at a dose of 90mg produced reduced duration ($p = 0.024$), incidence ($p = 0.009$) and severity ($p = 0.045$) of severe OM.	Superoxide dismutase mimetic. It rapidly converts O_2*— to H_2O_2, to arrest initiation of ROS cascade.

Supplementary Table S21. Summary of allopurinol as an intervention.

AUTHOR, YEAR	STUDY TYPE	POPULATION	INTERVENTION	COMPARATOR	OUTCOME	EFFECT OBSERVED	MECHANISM OF ACTION
Yokomizo et al., 2004 [88]	RCT	Patients with advanced or recurrent colon cancer<br>*N* = 52	Allopurinol ice 10mL<br>*N* = 20	No allopurinol ice<br>*N* = 32	Stomatitis was analysed via a grading system (not mentioned)	Allopurinol led to a decreased incidence and severity ($p = 0.0187$).	Inhibition of xanthine oxidase.

Supplementary Table S22. Summary of results of erythropoietin as an intervention.

AUTHOR, YEAR	STUDY TYPE	POPULATION	INTERVENTION	COMPARATOR	OUTCOME	EFFECT OBSERVED	MECHANISM OF ACTION
Hosseinjani et al., 2017 [89]	Double-blind RCT	Adults with non-Hodgkin's lymphoma, Hodgkin disease or multiple myeloma undergoing autologous HSCT $N = 80$	Erythropoietin mouthwash 50IU/ml $N = 40$	Placebo mouthwash $N = 40$	World Health Organization oral toxicity scale to measure OM incidence, duration, and severity	Significantly significant reduction in the incidence ($p < 0.001$) and duration ($p < 0.001$) of OM in intervention group.	Anti-inflammatory, antioxidant, and wound healing

Supplementary Table S23. Summary of results of glutamine as an intervention.

AUTHOR, YEAR	STUDY TYPE	POPULATION	INTERVENTION	COMPARATOR	OUTCOME	EFFECT OBSERVED	MECHANISM OF ACTION
Vidal-Casariego et al., 2013 [90]	Retrospective study	Patients treated with RT for H&N or cancer in chest area from 2008-2010 $N = 117$	Glutamine 30g/day received early (27.4%) or delayed (49.5%)	No glutamine (23.1%)	OM severity by WHO criteria	Risk difference of -9.0% (95% CI = -18.0% to -1.0%) for developing OM in the intervention compared to control group, and -14.0% (95% CI = -26.0% to -1.0%) for developing ARIE in intervention compared to control.	Antioxidant activity

Supplementary Table S24. Summary of results of N-acetyl cysteine as an intervention.

AUTHOR, YEAR	STUDY TYPE	POPULATION	INTERVENTION	COMPARATOR	OUTCOME	EFFECT OBSERVED	MECHANISM OF ACTION
Moslehi et al., 2014 [91]	Double blind RCT	Adult patients with AML, ALL, or myelodysplastic syndrome (MDS)	N-Acetyl Cysteine (NAC) 100mg/kg/day for 15 days $N = 38$	Placebo $N = 42$	OM incidence and grade via WHO oral toxicity scale,	OM incidence was not significantly different between groups. ($p = 0.34$)	NAC stimulates synthesis of glutathione,

		undergoing hematopoietic stem cell transfer (HSCT) with high-dose chemotherapy (HDC)			Glu.Px activity	Severe OM occurred less frequently in the NAC group to a significant extent (p = 0.04).<br>Mean duration of OM was significantly reduced in the NAC group. (p = 0.02)<br>Serum levels of Glu.Px were significantly higher in the NAC group 7 days following HSCT (p = 0.003).	scavenges free radicals.

Supplementary Table S25. Summary of results of propolis as an intervention.

AUTHOR, YEAR	STUDY TYPE	POPULATION	INTERVENTION	COMPARATO R	OUTCOME	EFFECT OBSERVED	MECHANISM OF ACTION
Salehi et al., 2018 [92]	Double blind, RCT	Patients with colon cancer being treated with folfox diet.<br>N = 50	50 mg propolis tablet, b.i.d. daily, for 21 days<br>N = 25	Placebo<br>N=25	OM incidence and grade via WHO oral toxicity	Significant difference in the mean severity of OM in the propolis group relative to the placebo group. (p = 0.027)<br>OM grade was significantly lower in the 2nd and 3rd follow up evaluations of the propolis group than the placebo group ($p < 0.05$).	Propolis previously demonstrated effective against oxidative stress in animal trials.

Supplementary Table S26. Summary of results of MF 5232 as an intervention.

AUTHOR, YEAR	STUDY TYPE	POPULATION	INTERVENTION	COMPARATOR	OUTCOME	EFFECT OBSERVED	MECHANISM OF ACTION
Naidu et al., 2005 [93]	Double-blind RCT	Patients with chemoradiation-induced OM	MF 5232, three times daily with food for 10-15 days	Placebo<br>N = 11	OM via WHO oral toxicity scale	Improvement in OM grade with intervention (p = 0.007).	MF 5232 has antioxidant activity.

		N = 22		*N* = 11			

Supplementary Table S27. Summary of results of melatonin as an intervention.

AUTHOR, YEAR	STUDY TYPE	POPULATION	INTERVENTION	COMPARATOR	OUTCOME	EFFECT OBSERVED	MECHANISM OF ACTION
Elsabagh et al., 2019 [94]	RCT	Head and neck cancer (HNC) patients receiving radiotherapy *N* = 40	Melatonin (20mg) per nocte for 6 weeks + symptomatic treatment *N* = 20	Symptomatic treatment (antifungals, topical anaesthetics, anti-inflammatories, topical analgesics, and sodium carbonate mouthwash) *N* = 18	OM via WHO oral toxicity scale<br>Discomfort and pain severity using the Numeric Rating Scale (NRS)<br>Total Antioxidant Capacity (TAC) via colorimetry	OM grading distribution was comparable between control and intervention, however there were fewer samples of higher grading in the intervention arm than the control.<br>Discomfort and pain (evaluated through the NRS) was lower for the melatonin group compared to the control. (p = <0.001)<br>TAC in the control group was reduced. (p = 0.004)	Melatonin a potent antioxidant capable of scavenging free radicals.

Supplementary Table S28. Summary of results of date palm pollen as an intervention.

AUTHOR, YEAR	STUDY TYPE	POPULATION	INTERVENTION	COMPARATOR	OUTCOME	EFFECT OBSERVED	MECHANISM OF ACTION
Elkerm et al., 2014 [95]	RCT	Patients with H&N cancer prior to exposure to first-line treatment *N* = 20	Date palm pollen 2g/day *N* = 10	Oral antifungal, rebamipide for mucosal restoration, and local and oral analgesics as needed *N* = 10	Assessment of oral tissues performed using OMAS, digital photographs and VAS	DPP associated with significantly (p-values unspecified) reduced OMAS score and pain severity of OM.	Blocks oxidative free radicals, prevents DNA damage, anti-inflammatory.

Supplementary Table S29. Summary of results of β-carotene as an intervention.

AUTHOR, YEAR	STUDY TYPE	POPULATION	INTERVENTION	COMPARATOR	OUTCOME	EFFECT OBSERVED	MECHANISM OF ACTION
Mills, 1998 [96]	RCT	Patients with advanced SCC of the mouth undergoing chemotherapy *N* = 20	Standard diet + beta-carotene supplement *N* = 10	Standard diet (no supplement) *N* = 10	OM via the Tygerberg Hospital Head and Neck Oncology Clinic OM grading system	In grade 3 & 4 OM, patients in the intervention arm developed severe OM later relative to controls. ($p < 0.025$) Reactions were less severe when compared to controls. ($p < 0.025$)	Beta-carotene is converted to vitamin A, a potent antioxidant.

Supplementary Table S30. Summary of results of calendula as an intervention.

AUTHOR, YEAR	STUDY TYPE	POPULATION	INTERVENTION	COMPARATOR	OUTCOME	EFFECT OBSERVED	MECHANISM OF ACTION
Babaee et al., 2013 [97]	RCT	H&N cancer patients *N* = 40	Calendula officinalis flower mouthwash 2% *N* = 20	Placebo mouthwash *N* = 20	Antioxidant capacity using Ferric reducing antioxidant power assay and oropharyngeal mucositis intensity using OMAS scale	Lower OMAS in intervention compared to placebo ($p = 0.031$).	Radical scavenging (hydroxyl groups), free radical terminators
